# Multiple versus single doses of dexamethasone in total hip arthroplasty: A protocol of randomized controlled trial

**DOI:** 10.1097/MD.0000000000020147

**Published:** 2020-05-08

**Authors:** Bangjian Zhang, Shaoqiong Sun, Bo Sheng

**Affiliations:** a Department of Anesthesiology; b Department of Operation room, Panzhihua Central Hospital; c Department of Anesthesiology, West China Second University Hospital, Sichuan University, Sichuan Province, China.

**Keywords:** dexamethasone, pain control, randomized controlled trial, study protocol, total hip arthroplasty

## Abstract

**Background:**

Reduction of post-operative pain, nausea, and vomiting in patients undergoing total hip arthroplasty (THA) may facilitate earlier discharge from hospital and reduce healthcare costs. The recommended dose regimen of dexamethasone in THA has not been determined. The purpose of this study was performed to compare the efficiency of multiple versus single doses of dexamethasone for early postoperative pain treatment after THA.

**Methods:**

This study was a randomized controlled trial which conducted in our hospital. Informed consent for participation in this trial was obtained from each patient before surgery. Two hundred patients undergoing THA received 1 dose of intravenous dexamethasone and 1 dose of normal saline (Group A), or 2 doses of intravenous dexamethasone (Group B). The primary outcome was visual analog scale pain scores in the immediate postoperative period. Secondary outcomes included postoperative opioid use, length of hospital stay, activity level during physical therapy, and hip range of motion.

**Results:**

This clinical trial might provide some insights to estimate the safety of dexamethasone.

**Trial registration:**

This study protocol was registered in Research Registry (researchregistry5460).

## 1 Introduction

Many multimodal regimens exist to minimize postoperative pain, nausea, and vomiting following total hip arthroplasty (THA). Postoperative nausea and vomiting is often cited as the most common adverse event after anesthesia, with a reported incidence of 20% to 83% after major orthopedic surgery.^[1]^ Despite an increased emphasis on postoperative pain control, multiple recent studies have indicated that 30% to 77% of patients experience moderate-to-severe pain postoperatively.^[2,3]^ Postoperative nausea and vomiting and inadequate pain relief can lead to significant discomfort, emotional distress, and lower patient satisfaction while interfering with physical therapy and prolonging hospitalization.^[4–6]^

Dexamethasone is a high-potency, long-acting glucocorticoid that has been extensively used in the perioperative setting.^[7]^ A single preoperative dose of dexamethasone is a proven prophylactic agent for nausea and vomiting. The analgesic and anti-inflammatory benefits of dexamethasone and other glucocorticoids have also been clearly demonstrated in anesthesia, general surgery, and orthopedic literature alike.^[8,9]^ However, concerns for potential side effects have prevented glucocorticoids from being regularly included in the perioperative protocols for THA despite randomized trials indicating short-dose glucocorticoids to be safe and effective for reducing postoperative pain and nausea.^[10]^

Nevertheless, due to clinical heterogeneity, the optimal timing, way, and dosage of dexamethasone in THA have not been clearly defined, which can lead to a great deal of variation in clinical results. In the study of Lei et al, the authors found that multiple low-dose dexamethasone can further relieve postoperative pain, ameliorate postoperative nausea, provide additional inflammatory control, enhance mobility, and shorten length of stay following THA.^[11]^ We, thus, also designed a randomized controlled study to compare the efficiency of multiple versus single doses of dexamethasone for early postoperative pain treatment after THA. Additionally, it was hypothesized that patients receiving multiple doses of dexamethasone would exhibit better postoperative outcomes compared with patients receiving single dose of dexamethasone.

## 2 Material and method

**Figure 1:**
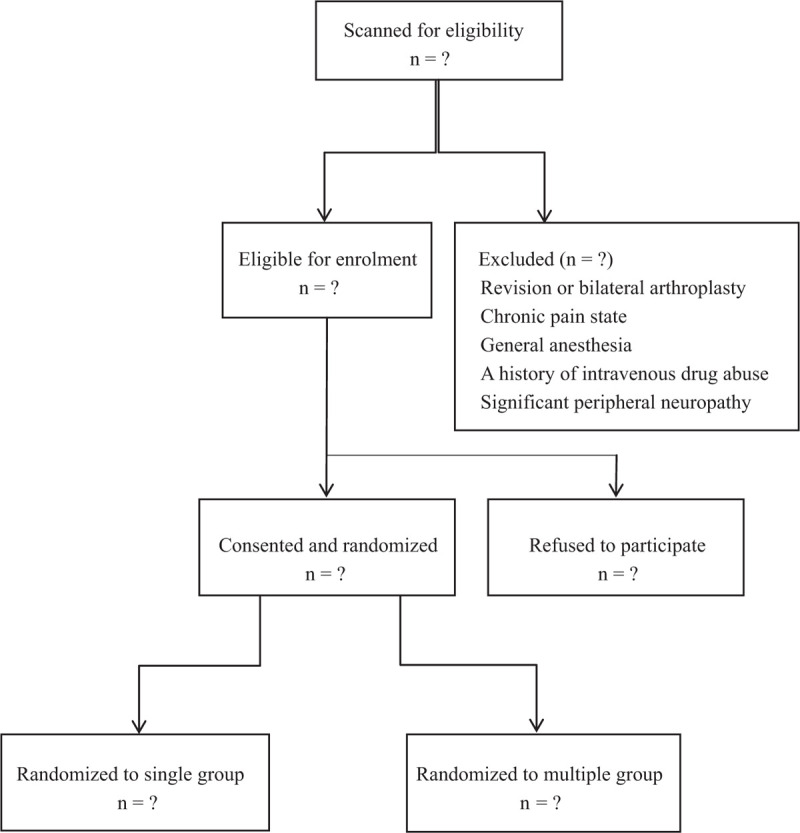
Flow diagram of the study.

This prospective, randomized, double-blind, controlled, superiority clinical trial was registered in Research Registry (researchregistry5460) and approved by the institutional review board in our university hospital (NC1001214). The conduct of this study followed the Declaration of Helsinki principles and the reporting of this study adhered to the Consolidated Standards of Reporting Trials guidelines for randomized controlled trials. The flowchart of this trial is shown in Figure 1.

### 2.1 Participants

We included male and female, American Society of Anesthesiologists physical status 1 to 4 patients, aged 18 to 85 years, weighing 50 to 100 kg, with a body mass index of 18 to 40 kg/m^2^, and undergoing elective primary, unilateral THA under spinal anesthesia. Exclusion criteria were revision or bilateral arthroplasty procedures, contraindications to spinal anesthesia, intraoperative general anesthesia, allergy to any of the study medications, chronic pain state or opioid dependence unrelated to hip pathology (defined as ongoing requirement of opioids for analgesic management of any ongoing pain state that is unrelated to hip pathology for longer than 12 weeks), and significant peripheral neuropathy. Most patients were identified and screened by a research assistant at the time of their pre-admission visit, provided the details of the clinical trial procedures, and given time to consider enrollment. Some patients were consented on the day of surgery, but identical consent procedures were observed.

### 2.2 Randomization and blinding

After written informed consent was taken, patients were randomly allocated to one of the two groups in a 1:1 ratio using a computer-generated list of random numbers with a block randomization technique (www.randomization.com) by a research assistant. A unique randomization code was used with no restrictions to either of the two study groups: single dose or multiple doses dexamethasone. The results of the randomization were maintained in opaque envelopes and stored with the research coordinator. These sealed, opaque envelopes were given to the operating room nurse who then prepared the single or multiple doses dexamethasone according to the randomization code. This operating room nurse had no further participation in any aspect of the study. The patient, anesthesiologist, surgeon, physiotherapists, acute pain nurses, and outcome assessors were unaware of study group allocation.

### 2.3 Perioperative management

All patients received 650 to 1000 mg acetaminophen orally, adjusted to weight and 100 to 200 mg celecoxib before surgery unless contraindicated. All patients received spinal anesthesia in a designated regional anesthesia room. After placement of non-invasive blood pressure, ECG, and pulse oximetry monitors, as well as a facemask with supplemental oxygen and intravenous access, patients were then positioned in the sitting position, and their backs were prepared with 2% chlorhexidine gluconate in 70% isopropyl alcohol for spinal anesthesia.

In the operating room, patients underwent THA in the lateral decubitus position and received oxygen via facemask to maintain oxygen saturation of more than 92%, unless otherwise indicated. No patients received intravenous dexamethasone at any time during the perioperative period. All other perioperative procedures adhered to standard institutional practices. All THA procedures were performed by fellowship-trained, high-volume surgeons. All patients underwent a standard lateral approach THA with uncemented implants, with division of the abductor medius and minimus to access the joint. All tendon divisions were repaired before wound closure. Group A served as the single group and received 1 dose of intravenous dexamethasone and 1 dose of normal saline; Group B received 2 doses of intravenous dexamethasone. The first dose was administered by analgesists immediately prior to anesthesia induction, and the second dose was administered by a nurse immediately upon the patient's return to the inpatient unit (3–4 h after the first dose).

### 2.4 Outcome measures

The primary outcome was visual analog scale (VAS) pain scores in the immediate postoperative period (postoperative day 0 through 3). VAS scores were recorded by nursing staff, blinded to treatment group, every 6 h throughout the hospital stay. VAS scores on each postoperative day were averaged, and the daily averages were used for analysis. Secondary outcomes included postoperative opioid use, length of hospital stay, activity level during physical therapy, and hip range of motion. Total opioid consumption was calculated by converting opioids consumed to morphine equivalents. Length of hospital stay was calculated by measuring the time from the completion of surgery through discharge for each patient. Activity level during physical therapy was recorded by measuring the steps taken in daily physical therapy sessions. Hip range of motion was measured by the surgeon using a goniometer in the office at 3 weeks postoperatively.

### 2.5 Sample size calculation

The sample size was determined for the primary endpoint and was calculated using PASS 2011 software (NCSS, LLC, Kaysville, UT). According to the results of our previous study, the postoperative VAS score for nausea was 2.16 in the control group. We anticipated a difference of 0.72 in the VAS score. With a power of 0.90 and significance level of 0.05, the required sample size was calculated as 50 in each arm. Considering possible exclusion, we decided to include 80 patients in each group.

### 2.6 Statistical analysis

All statistical analyses are performed using SPSS v. 24 (IBM Corp., Armonk, NY). Descriptive statistics of demographic and clinical characteristics are presented with mean standard deviation for continuous scale variables. The difference between normally distributed continuous scale variables is examined using Student's *t* test, while nonnormal variables are examined using Wilcoxon rank sum test. The association between categorical variables is examined using Pearson Chi-squared test or Fisher's exact test. All analyses are performed in accordance with intention-to-treat principle.

## 3 Discussion

Immediately after a surgical incision, a range of inflammatory, metabolic, hormonal, and immune responses are activated as part of the overall stress reaction. Afferent neuronal activation of the hypothalamic–pituitary–adrenal axis sets off cascades of reactions resulting in the release of cortisol within minutes, reaching a maximum 4 to 6 h later.^[12]^ This stress response may have a host of negative effects on the post-surgical patient, including pain, nausea, poor sleep, and dissatisfaction.^[13]^ Corticosteroids, such as dexamethasone, exert a powerful anti-inflammatory effect via molecular mechanisms acting at the level of transcription initiation. Corticosteroids are not without harmful side effects, which include increased risk of thromboembolism, gastric ulceration, hyperglycemia, and wound complications.^[14,15]^

In THA, however, the ideal dosage and route of dexamethasone administration is unclear. Consequently, we performed this study to clarify the clinical effect and safety of a multiple-dose dexamethasone regimen. This trial has some limitations. First, the subjects may be exclusively Chinese. Therefore, the data from this clinical trial cannot be applied to other ethnic groups. Second, owing to the small sample size, the results of this study cannot be generalized. Despite these limitations, this clinical trial might provide some insights to estimate the safety of dexamethasone.

## Author contributions

**Conceptualization:** Shaoqiong Sun.

**Data curation:** Bangjian Zhang.

**Formal analysis:** Bangjian Zhang, Shaoqiong Sun.

**Funding acquisition:** Bo Sheng.

**Investigation:** Bangjian Zhang, Shaoqiong Sun.

**Methodology:** Bangjian Zhang, Shaoqiong Sun.

**Resources:** Bo Sheng.

**Software:** Bangjian Zhang, Shaoqiong Sun.

**Supervision:** Bo Sheng.

**Validation:** Bo Sheng.

**Visualization:** Shaoqiong Sun.

**Writing – original draft:** Bangjian Zhang.

**Writing – review & editing:** Shaoqiong Sun, Bo Sheng.
